# Improving Physical Activity in Adults Admitted to a Hospital With Interventions Developed and Implemented Through Cocreation: Protocol for a Pre-Post Embedded Mixed Methods Study

**DOI:** 10.2196/19000

**Published:** 2020-11-13

**Authors:** Sven J G Geelen, Boukje M Giele, Frans Nollet, Raoul H H Engelbert, Marike van der Schaaf

**Affiliations:** 1 Department of Rehabilitation Medicine Amsterdam UMC, University of Amsterdam Amsterdam Movement Sciences Amsterdam Netherlands; 2 Center of Expertise Urban Vitality Faculty of Health Amsterdam University of Applied Sciences Amsterdam Netherlands

**Keywords:** implementation science, quality improvement, physical activity, mobility, outcome and process assessment, health care

## Abstract

**Background:**

Admission to a hospital is often related with hospital-associated disabilities. Improving physical activity during hospitalization is considered effective to counteract hospital-associated disabilities, whereas many studies report on very low physical activity levels. Gradually developing and implementing interventions in cocreation with patients and health care professionals rather than implementing predefined interventions may be more effective in creating sustainable changes in everyday clinical practice. However, no studies have reported on the use of cocreation in the development and implementation of interventions aimed at improving physical activity.

**Objective:**

This protocol presents a study that aims to investigate if interventions, which will be developed and implemented in cocreation, improve physical activity among patients in surgery, internal medicine, and cardiology hospital wards. The secondary aims are to investigate effectiveness in terms of the reduction in the time patients spend in bed, the length of hospital stay, and the proportion of patients going home after discharge.

**Methods:**

The Better By Moving study takes place for 12 months at the following five different wards of a university hospital: two gastrointestinal and oncology surgery wards, one internal medicine hematology ward, one internal medicine infectious diseases ward, and one cardiology ward. The step-by-step implementation model of Grol and Wensing is used, and all interventions are developed and implemented in cocreation with health care professionals and patients. Outcome evaluation is performed across the different hospital wards and for each hospital ward individually. The primary outcome is the amount of physical activity in minutes assessed with the Physical Activity Monitor AM400 accelerometer in two individual groups of patients (preimplementation [n=110], and 13 months after the start of the implementation [n=110]). The secondary outcomes are time spent in bed measured using behavioral mapping protocols, and length of stay and discharge destination assessed using organizational data. A process evaluation using semistructured interviews and surveys is adopted to evaluate the implementation, mechanisms of impact, context, and perceived barriers and enablers.

**Results:**

This study is ongoing. The first participant was enrolled in January 2018. The last outcome evaluation and process evaluation are planned for May and June 2020, respectively. Results are expected in April 2021.

**Conclusions:**

This study will provide information about the effectiveness of developing and implementing interventions in cocreation with regard to improving physical activity in different subgroups of hospitalized patients in a university hospital. By following step-by-step implementation and by performing process evaluation, we will identify the barriers and enablers for implementation and describe the effect of new interventions on improving physical activity among hospitalized patients.

**Trial Registration:**

Netherlands Trial Register NL8480; https://www.trialregister.nl/trial/8480

**International Registered Report Identifier (IRRID):**

DERR1-10.2196/19000

## Introduction

Admission to a hospital is often related with the occurrence of hospital-associated disabilities, such as a reduced muscle mass, reduced muscle strength, malnutrition, and new limitations in activities of daily living (ADLs) [1-3]. In turn, hospital-associated disabilities are related with a prolonged length of stay, increased risk of institutionalization, permanent loss of ADLs, and mortality [4-6]. As hospital-associated disabilities are frequently registered in hospitalized older patients [7] and the age of the general population increases by the year, it is important to develop intervention strategies to reduce hospital-associated disabilities.

Improving physical activity during hospitalization is considered to be effective for counteracting hospital-associated disabilities [1,8-10]. Several studies showed that early mobilization and increasing physical activity in surgical and nonsurgical patients reduces hospital length of stay and improves independence in daily activities and discharge destination [11-13]. Yet, despite the knowledge that increasing physical activity contributes to the prevention of in-hospital functional decline, many studies continue to report on very low physical activity levels among hospitalized patients [14,15].

Previous research showed that physical activity in specific subgroups (ie, gastrointestinal surgery, internal medicine, and stroke) of hospitalized patients can be improved with a single intervention involving a one size fits all approach [10,16,17]. Gradually developing and implementing interventions in cocreation rather than implementing predefined interventions is believed to be more effective in creating sustainable changes in everyday clinical practice [18-20]. However, no studies have recently reported on the use of cocreation in the development and implementation of interventions aimed at the improvement of in-hospital physical activity. Therefore, the Better By Moving study in our university hospital is the first study that has been developed to investigate whether interventions, which will be developed and implemented in cocreation with patients and health care professionals, improve physical activity in patients admitted to surgery, internal medicine, or cardiology hospital wards. Moreover, by improving physical activity, we aim to reduce the time patients spend in bed, reduce hospital length of stay, and improve the number of patients going home after discharge. A systematic process evaluation provides important information on barriers and facilitators for future quality improvement projects aiming to improve physical activity in hospitalized patients.

## Methods

### Study Design

An uncontrolled pre-post embedded mixed-methods study is designed to evaluate whether we can improve physical activity in hospitalized patients by using interventions that we develop and implement in cocreation. The development and implementation describe the iterative (cyclical) process of planning, conducting, reflecting, and refining, which is being used in close collaboration with local stakeholders, such as patients, health care professionals, and managers [19]. The study has been approved by the Medical Research Ethics Committee of the Amsterdam University Medical Centers (Amsterdam UMC), Academic Medical Center (W17_479 #18.003 and W19_213 #19.258). Written informed consent will be obtained from all participants in both the outcome and process evaluations.

### Setting

This study will be conducted at five different hospital wards (two gastrointestinal and oncology surgery wards, one internal medicine hematology ward, one internal medicine infectious diseases ward, and one cardiology ward) in a 1000-bed tertiary university teaching hospital Amsterdam UMC, Academic Medical Center, the Netherlands. Each hospital ward has 29 beds and a nursing-to-patient ratio of either 1:3 or 1:4, depending on the patient acuity. Allied health staffing involves 0.5 to 1 physical therapists for each hospital ward.

### Development and Implementation of Interventions

**Figure 1:**
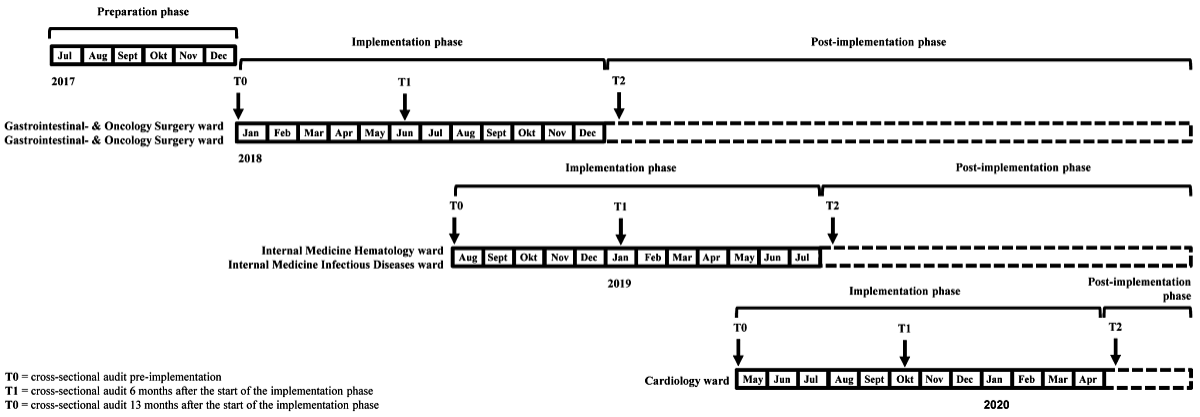
Project timeline.

The Better By Moving study consists of a 6-month preparation phase and a 12-month hospital ward-specific implementation phase, starting in January 2018. The entire project timeline has been described in Figure 1. At each hospital ward, the step-by-step implementation model of Grol and Wensing will be used [21]. A summary of the different steps according to Grol and Wensing has been described below. Stakeholders participate in cocreation at the following different levels as described by Cornwall: “co-option,” “compliance,” “consultation,” “co-operation,” “colearning,” and “collective action” [22,23].

#### Step 1: Defining the Proposal for Change

The purpose of step one is to finalize the Better By Moving project plan. Therefore, hospital-wide attention is attracted on the benefits of physical activity with presentations and workshops during the 6 months prior to the start in the first hospital ward. Patient representatives, local stakeholders (ie, nurses, physicians, rehabilitation professionals, managers, and team leaders), and experts working on this topic in different hospitals will be asked to participate in discussions to develop the project plan.

#### Step 2: Analysis of Actual Performance

The purpose of step two is to quantify the outcome measures at baseline. Therefore, a cross-sectional audit will be performed at each hospital ward prior to assess the total amount of physical activity using accelerometers (Physical Activity Monitor [PAM] AM400, PAM BV) [24,25]. In addition, behavioral mapping protocols [26-28] will be used during the same cross-sectional audits to assess the time patients spend in bed, as well as on other physical activities. While the PAM AM400 accelerometer assesses the activity duration and intensity by measuring accelerations, the behavioral mapping protocols indicate how much time patients spend on each type of activity (ie, lying in bed, sitting, standing, or walking) by observing the patient every 10 minutes. Both outcomes complement each other in the understanding of in-hospital physical activity behavior. Further details on both assessments and the included patient population can be found in “Outcome and Process Evaluation.”

#### Step 3: Analysis of Barriers and Enablers Among Patients and Health Care Professionals

The purpose of step three is to gain insights into the barriers and enablers to improve physical activity during hospital stay. Barriers and enablers for physical activity as perceived by patients and health care professionals will be assessed by a mixed-methods design using surveys, interviews, observations, and focus group discussions.

The patient surveys identify the perceived barriers and enablers to physical activity using two open-ended questions, the level of encouragement patients perceive from health care professionals and context using six questions with a 5-point scale based on the questions of van Delft et al [29], and their perceived self-efficacy in performing basic mobility activities independently using seven standardized questions with a 5-point scale based on the Short Falls Efficacy Scale-International [30]. Patients participating in the baseline cross-sectional audit will be asked to complete the survey. In addition, to further assess the perceived barriers and enablers to physical activity, patients will be asked to participate in an additional short face-to-face interview using the following purposeful sampling criteria: survey responses and age.

To identify the barriers and enablers as perceived by health care professionals, we developed a survey based on the theoretical domains framework (TDF) [31]. The TDF encompasses 12 domains, providing a theoretical lens to view all cognitive, affective, social, and environmental influences on behavior and behavior change. Using the 12-domain TDF as a basis, a multidisciplinary team of physical therapists, senior researchers, nurses, and a medical psychologist developed a survey consisting of 39 items and a 5-point Likert scale (totally disagree, disagree, neutral, agree, and totally agree), with two items assessing the health care professional’s “knowledge” with regard to improving physical activity in hospitalized patients, one item assessing the health care professional’s “skills,” one item assessing “social/professional role and identity,” two items assessing “beliefs about capabilities,” five items assessing “beliefs about consequences,” two items assessing “motivation and goals,” one item assessing “memory, attention, and decision processes,” 17 items assessing “environmental context and resources,” two items assessing “social influences,” two items assessing “emotion,” three items assessing “behavioral regulation,” and one item assessing “nature of behaviors.” The TDF ensures that all cognitive, affective, social, and environmental influences on behavior will be considered. Further elaboration on the 12 domains of the TDF can be found in the study of Atkins et al [31]. The survey will be distributed among all health care professionals working in each of the hospital wards. Subsequently, at each hospital ward, focus groups will be held to further substantiate the most relevant items. Participants will be asked to participate in the focus groups based on the following purposeful sampling criteria: survey responses, age, working experience, and profession. Finally, health care professionals will be observed at random intervals during the 12-month implementation phase to better understand the daily hospital care, culture, environment, and context (ie, social and environmental influences) in each of the hospital wards.

#### Step 4: Development and Selection of Interventions and Strategies

The purpose of step four is to develop interventions and strategies in cocreation while taking into account the barriers and enablers raised by hospitalized patients and health care professionals. Therefore, a working group will be formed at each hospital ward with the project manager (SJGG), nurses, physicians, and a physical therapist. In periodic working group meetings, interventions most suitable to the local context will be developed based on information from the previous steps. Through the use of an iterative (cyclical) process of planning, conducting, reflecting, and refining, the working group will develop various interventions [19]. The behavioral change wheel (BCW) framework will be used to guide the development of interventions [32]. In the BCW, behavior is explained as part of an interacting system between capability, opportunity, motivation, and behavior, also known as the COM-B model. These BCW components will help the working groups to better understand the patients’ and health care professionals’ behaviors. Moreover, the use the BCW framework will help the working groups to identify optimal behavioral change techniques, which they can incorporate in the detailed intervention proposals [32]. Working group progress will be closely coordinated and supported by the project manager, and the project manager will keep track of the cocreation process using an audit trail. When needed for the iterative (cyclical) development process, the working groups will consult caregivers, family members, patient representatives, local stakeholders, hospital managers, or experts regarding in-hospital physical activity in different hospitals for additional input. At random intervals, a group of patients from the hospital ward will be asked for feedback on the interventions.

#### Step 5: Development, Testing, and Execution of an Action Plan With Multiple Interventions

The purpose of step five is to gradually implement the intervention proposals in the local context. For each intervention proposal, a testing and implementation plan will be developed in collaboration with the local hospital ward team leader and carried out by the hospital ward specific working group. Hospital managers will be involved and will provide input on a regularly basis. Experience with potentially effective interventions will be translated to the subsequent participating hospital wards.

#### Step 6: Including Integration of Changes in Routine Care

The purpose of step 6 is to ensure implemented interventions are integrated in routine hospital care. All implemented interventions considered potentially effective will therefore be further refined by the working group and project manager during the 12-month implementation phase. In consultation with hospital managers, local team leaders, and the working group, integration in daily hospital practice will be ensured. In addition, tools will be developed for each hospital ward to systematically evaluate the implementation of the interventions.

### Outcome and Process Evaluation

#### Target Population

A cross-sectional audit will be conducted at baseline, 6 months, and 13 months after the start of the implementation phase. During each cross-sectional audit, a random sample of hospitalized patients will be approached to participate during one day from 8 AM to 8 PM. Eligible patients are 18 years or older, have sufficient Dutch or English speaking ability and reading skills, and are admitted for at least 24 hours. The following exclusion criteria will be used: inability to perform independent transfers prior to hospital admission, delirium, obligatory bed rest as indicated by the attending physician, expectation to be discharged before 12 AM on the day of observation, and receiving end-of-life care. Random selection of potential participants will be performed using a computer-generated list based on the room numbers of the hospital ward, and potential participants will be approached one or two days prior to the day of observation. In the case of refusal or when the patient does not meet the study criteria, the investigator will approach the patient in the next hospital room on the computer-generated list. Each participant can only be enrolled once. No a priori sample size calculations are performed. Resources allow us to spend 1 year at the iterative (cyclical) process at each hospital ward; therefore, we determined a pragmatic sample size. Considering the duration of the different steps, including three cross-sectional audits, the inclusion of 65 participants is deemed feasible at each hospital ward. Given this sample size (n=110 at baseline and n=110 at 13 months) and assuming normality of the outcome parameter, we will be able to detect an effect size of 0.38 or higher for the primary outcome (two groups *t* test of equal means; α=.05; 1−β=.80; nQuery 8, Statistical Solutions Ltd).

#### Primary Outcome

The primary outcome is the total amount of physical activity in minutes (>1.4 metabolic equivalent tasks [METs] [33]), which will be measured during each cross-sectional audit from 8 AM to 8 PM using the PAM AM400 wireless accelerometer. The PAM AM400 is a 2-cm-wide coin and is waterproof, and it will be attached to the ankle. The PAM AM400 measures accelerations 10 times per second in three dimensions and converts these accelerations to the total amount of time of physical activity in minutes >1.4 METs. METs is a concept that is used to assign an intensity value to specific activities. In healthy participants, light-intensity physical activity involves <3.0 METs, moderate physical activity involves 3.0-6.0 METs, and vigorous physical activity involves >6.0 METs [33]. Sedentary behavior is defined as ≤1.5 METs [34]. In addition to the total amount of physical activity in minutes >1.4 METs, the PAM AM400 compares each second of physical activity with the following three predefined intensity zones: light physical activity (1.4-3.0 METs), moderate physical activity (3.0-7.0 METs), and vigorous physical activity (>7.0 METs), and measures the derivative of METs for 24-h physical activity (PAM score = [METs − 1] × 100 averaged over the day). The validity and reliability of the PAM in healthy adults is moderate to good for assessing the estimate of energy expenditure [24,25].

#### Secondary Outcomes

Secondary outcomes include the time patients spend in bed, length of stay, and discharge destination. Data on the time patients spend in bed will be measured during each of the cross-sectional audits using the behavioral mapping method [26-28]. In detail, structured observations will be undertaken by trained physical therapy graduate students for a 1-minute period every 10 minutes between 8 AM and 8 PM, using a predetermined set of mutually exclusive types of activities (lying in bed, sitting on the edge of a bed or sitting in a chair, making a transfer from bed to chair, or standing, walking, and using the ergometer). For each minute of observation, the activity with the highest intensity is recorded. Patients are not followed off the ward and not intruded on if behind closed curtains. In addition, the following patient characteristics will be collected during each of the cross-sectional audits: sex, age, comorbidities, number of functional restraints (eg, intravenous lines and drains), functional ability with the Katz-ADL 2 weeks before admission [35], and independence in mobility using the Activity Measure for Post-Acute Care “six clicks” Basic Mobility Short Form [36]. Data will be directly recorded in the online Castor Electronic Data Capture database (Ciwit BV).

Data on length of stay and discharge destination will be obtained from the hospital administrative data for all patients admitted to the surgery, internal medicine, and cardiology hospital wards. Discharge destination will be categorized as follows: going home or going to a temporary institution (ie, nursing home, geriatric rehabilitation center, or medical rehabilitation center). Data on patients who are discharged to a permanent nursing home or other hospitals, those who receive end-of-life care (at home or at a facility), or those who die during hospitalization will be omitted because other influences (such as illness, prognosis, and cognitive function) determine the outcome.

#### Process Evaluation

Process evaluations are advised to monitor the implementation processes of complex interventions. In this study, the framework of the Medical Research Council guideline is followed to guide the process evaluation [31,37]. The three key functions of this framework include “implementation,” “mechanisms of impact,” and “context.” “Implementation” contains the goals and interventions that have been delivered, and how the implementation is achieved. The “mechanisms of impact” include the response to the interventions, the mediators, and all (unexpected) results and consequences. “Context” includes all other factors that may affect the implementation, interventions, and outcomes, such as barriers (eg, openness to changes, motivation, workload, and costs) and enablers [31]. In this study, we will assess these three key functions by using semistructured interviews by purposefully selecting health care professionals, team leaders, and managers 13 months after the start of the implementation. A topic guide in Dutch will be developed specifically for each hospital ward, which will consist of items covering all three key functions. In addition, 13 months after the start of the implementation, we will re-evaluate the perceived barriers and enablers to physical activity, the level of encouragement patients perceive from health care professionals and context, and their perceived self-efficacy in performing basic mobility activities independently using the survey described in step 3. We will assess the experience with our implemented interventions and various aspects of implementation fidelity (ie, adherence, exposure, and participant responsiveness) by adding both questions with a 5-point scale and open-ended questions to the patient survey (eg, “Did you receive …?” and “If so, did you find … of added value?”). We will also assess health care professionals’ perception and experience with the project and our implemented interventions by using an additional survey with open-ended questions (eg, “The following intervention does/does not add to more in-hospital physical activity …” and “Were you made aware of …?”) and by adding specific questions to our process evaluation topic guide.

### Data Analysis

All analyses will be conducted using IBM SPSS Statistics version 25 (IBM Corp). Patients’ characteristics in each cross-sectional audit will be described using descriptive statistics. Primary outcome evaluation will be performed between month 0 and month 13 across all hospital wards, and only the data of patients wearing the PAM during the entire observation period (8 AM-8 PM) will be used. The total amount of physical activity in minutes will be tested on normality with the Kolmogorov-Smirnov test and will be visually inspected using Q-Q plots. A logarithmic transformation will be considered in case of nonnormality. Analysis of covariance will be used to assess the difference in the total number of minutes of physical activity (>1.4 METs), whereby the covariates include independence in mobility and the presence of a urinary catheter. Both covariates are based on unpublished results of multivariable regression models considering various patient factors in relation to physical activity. In case nonnormality persists after logarithmic transformation, a Poisson regression model will be considered using the same covariates.

Additionally, data of behavioral mapping observations will be categorized into different activity types, from which time spent lying in bed between 8 AM and 8 PM in percentage will be derived. The difference in time patients spend lying in bed will be assessed among months 0, 6, and 13. An interrupted time series (ITS) will be used to evaluate the changes in length of stay and discharge destination among the following three predefined periods: 12 months prior to the implementation phase, 12 months implementation phase, and 6 months after finishing the implementation phase [38].

For the process evaluation, MAXQDA Analytics Plus 2018 (VERBI Software) will be used to facilitate the data analysis. All semistructured interviews will be thematically analyzed following the methods of Braun and Clarke [39]. The analytic process will be performed by two independent researchers (BMG and SJGG) and supervised by MvdS. Consensus meetings will be used to discuss and refine each theme. Member checking will be used to ensure the credibility of the data analysis. Triangulation of data will be performed by using the open-ended survey data during the qualitative data collection and analysis. Patient and health care professional survey results will be compared using chi-square tests and analysis of variance tests, depending on the type of data.

## Results

This study is ongoing. The first participant was enrolled in January 2018 at the gastrointestinal and oncology surgery ward. The last outcome evaluation and process evaluation are planned for May and June 2020, respectively. Results are expected in April 2021. A summary of all participation types within this study can be found in Multimedia Appendix 1.

## Discussion

While the amount of evidence on the negative consequences of physical inactivity during hospitalization continues to grow, few studies have evaluated the effectiveness of interventions that have been specifically tailored (ie, developed and implemented) in collaboration with the target population. So far, several studies revealed that increasing physical activity in general or encouraging early mobilization (after admission or operation) has a positive influence on physical functioning in daily activities, the duration of admission, and discharge home [11,13]. However, these studies often focus on unilateral interventions and have been performed in a specific context, while physical inactivity seems to affect patients in all hospital wards and patients of all ages [27]. The integration of multiple interventions in daily hospital care entails various challenges, as also described in the quality improvement projects of Mudge et al and Hoyer et al [9,16]. The Eat Walk Engage program of Mudge et al describes an approach using multiple interventions, which demonstrated a reduced length of stay after implementation in older hospitalized patients [9]. In addition, the currently ongoing Hospital in Motion study of van Delft et al describes the usage of multiple interventions tailored to tackle the numerous described barriers perceived by health care professionals and hospitalized patients [29]. The Better By Moving study will contribute to this research by providing more insight into the effectiveness of interventions that are developed bottom-up and in cocreation with the target population and by thoroughly analyzing the process of cocreation.

The strength of the Better By Moving study is the thorough problem analysis of actual performance, and barriers and enablers, which will be carried out prior to the development of the first intervention. More specifically, the barriers and enablers perceived by patients or health care professionals will be assessed through different mixed methods, such as surveys, physical measurements, observations, interviews, and focus groups. In addition, we hypothesize that the extensive analysis will create support among health care professionals, manifest ownership among local stakeholders, and facilitate the development of a local testing and implementation plan. Second, colearning in the development and implementation of new interventions together with local stakeholders from five different hospital wards can offer both an in-depth and a broad perspective on what works and what does not work when trying to improve physical activity in hospitalized patients. Third, the use of evidence-based behavioral change theories, such as the TDF [31] and BCW [32], makes it more likely that the underlying reasons for physical inactivity in hospitalized patients will be identified and countered [31,32]. Finally, the ITS analysis, which will be used, is considered one of the strongest quasiexperimental designs to evaluate outcomes such as length of stay and discharge destination. So far, none of the previously published studies investigating physical activity–improving interventions incorporated ITS analysis.

Diverse factors could challenge the success of the Better By Moving study. First, several “system” factors may affect the implementation process, such as a change in the environmental context (ie, staff turnover, competing trials, and workload) and the hospital ward culture (ie, attitude to change, commitment, and motivation) [40]. For instance, the planned renovation of the participating hospital wards and the recent merger with the Vrije Universiteit Medical Center may create a lack of focus and provide additional workload. Second, because it is not known in advance which interventions will be developed and implemented, the achieved effect may differ in each hospital ward owing to differences in the interventions used. To counter this as much as possible, we will provide for both an overall and a ward-specific analysis. Third, changing the health care professionals’ and patients’ behaviors toward in-hospital physical activity through the development and implementation of multiple interventions in cocreation takes time. While we have 12 months for each hospital ward to cocreate interventions, important changes in hospital culture, environmental context, and outcomes may arise after the last cross-sectional audit. Lastly, a pre-post mixed-methods design is used to investigate if interventions developed and implemented in cocreation improve physical activity among patients. To evaluate the effect of an intervention, a randomized controlled trial is considered the gold standard. With respect to cocreational bottom-up intervention development and implementation, in which the process, to a large extent, determines the outcome, it is considered not feasible to use a control group. Instead, we aim to evaluate the impact of our interventions on physical activity as representative as possible by approaching a random sample of at least 110 hospital patients using a computer-generated list based on hospital room numbers before and after the implementation of interventions.

By using cocreation to develop and implement interventions and by performing a process evaluation, useful insights will be provided on the effect and underlying processes of bottom-up intervention development and implementation in close collaboration with the target population and local stakeholders. Using this information, health care professionals, managers, and researchers will be able to better assess the elements that do and do not work with regard to improving physical activity in daily hospital care.

## Appendix

Multimedia Appendix 1 Data sources and participation types used for intervention development.

## Abbreviations

ADLs: activities of daily living
BCW: behavioral change wheel
ITS: interrupted time series
MET: metabolic equivalent task
PAM: Physical Activity Monitor
TDF: theoretical domains framework
UMC: University Medical Centers
