# Supplementary material for: Improving Physical Activity in Adults Admitted to a Hospital With Interventions Developed and Implemented Through Cocreation: Protocol for a Pre-Post Embedded Mixed Methods Study
Source: JMIR Res Protoc. 2020 Nov 13;9(11):e19000. doi: 10.2196/19000 (PMC7695526; doi:10.2196/19000)
Supplement: Multimedia Appendix 1 [file resprot_v9i11e19000_app1.pdf]

This is a Multimedia Appendix to a full manuscript published in the J Med Internet Res. For full copyright and citation information see http://dx.doi.org/10.2196/jmir.1900

Table S1. Data sources and types of participation used for intervention development.

Data source	Purpose	Data type	Description	Type of collaboration[a]	Sample	Time point
Local stakeholders (i.e. managers, team leaders)	A stakeholder analysis will be carried out to map the various interests and relationships; Providing regular feedback.	Brainstorm; Review; Discussion	Evaluation of the project plan; Discuss intervention proposals; Discuss implementation strategies	Co-operation; Consultation	Nursing manager, team leaders, officer 'Quality and Safety', board of directors	Every 3 months during the entire duration of the project; Occasionally depending on the interventions
National group of experts focusing on improving physical activity in hospitalized patients	Keeping abreast of national developments in the field of improving physical activity during hospitalization	Brainstorm	Discussing results of similar projects; Discuss intervention proposals; Discuss implementation strategies	Consultation	Project managers, researchers and physical therapist involved in projects improving physical activity in the Netherlands	Every 3 months during the entire duration of the project; Occasionally depending on the interventions
Client council (patient representatives)	To give advice through patient perspective	Presentation of ideas, results etc.	Keeping informed about the project and ask for input	Consultation	Five patient representatives of the local counsel	T0, T1, T2
Patients	To gain insight in the physical activity behavior of patients.	Participant observation	Cross-sectional audits using wireless accelerometers (Physical Activity Monitor AM400) from	Co-option	At least 22 randomly approached clinically admitted patients per included hospital ward	T0, T1, T2

			8 AM to 8 PM			
Patients	To gain insight in the amount of time patients spent in bed;<br>To get insight in the physical activity behavior of patients.	Participant observation	Cross-sectional audits using behavioral mapping protocols from 8 AM to 8 PM	Co-option	At least 22 randomly approached clinically admitted patients per included hospital ward	T0, T1, T2
Patients	To identify the perceived barriers and enablers to physical activity, the level of encouragement patients perceive from the health care professionals and context, and their perceived self-efficacy in performing basic mobility activities independently	Survey	Standardized surveys with 18 questions, consisting of three open-ended questions and 15 questions with a 5-point Likert-scale (totally disagree, disagree, neutral, agree, totally agree)	Co-option	At least 22 randomly approached clinically admitted patients per included hospital ward	T0, T1, T2
Patients	To identify barriers and enablers physical activity in patients admitted to the specific hospital ward	Short face-to-face semi-structured interviews	Short face-to-face interviews, using the results of the standardized questions	Co-option	At least 22 randomly approached clinically admitted patients per included hospital ward	T0, T1
Patients	To provide the working group with new ideas<br>To provide new intervention proposals with input;<br>To provide testing-and-implementation plans with input	Brainstorm; Review	Short face-to-face interviews, using the results of the standardized questions<br>Discuss testing-and-implementation plans	Consultation	Randomly approached health care professionals working on the hospital wards	At random intervals during the implementation phase

Health care professionals	To identify barriers and enablers involved in improving the patient's physical activity	Survey	TDF-based checklist with 39-questions: encompasses 12 domains, representing factors from psychological and organizational behavioral change theories.	Co-option	All health care professionals working on the hospital wards	T0, T2
Health care professionals	To identify key barriers and enablers for changing behavior	In-depth, semi-structured focus groups	Discuss the most relevant determinants.	Compliance	Voluntary participation of a multidisciplinary group, 6-10 persons per hospital ward.	T0
Health care professionals	To gain insight in the working process of the health care professionals	Participant observation	Observing daily hospital care, culture, environment and context	Co-option	Randomly approached health care professionals working on the hospital wards	T0, T1, T2 and at random intervals
Working group	To identify feasible and effective interventions, develop intervention proposals, develop testing-and-implementation plans and implement given intervention proposals	Brainstorm	Identifying feasible and effective interventions; Develop intervention proposals; Develop testing-and-implementation plans; Implementing intervention proposals	Co-learning	The project manager, and nurses, physicians and a physical therapist of each hospital ward	A meeting every 2 to 3 weeks during the implementation phase; Occasionally depending on the iterative process

TDF = Theoretical Domains Framework; T0 = cross-sectional audit preimplementation; T1 = cross-sectional audit 6 months after start of implementation phase; T2 = cross-sectional audit 12 months after start of implementation phase; [a]Cornwall's participation levels [23]: ''Co-option": where token representatives are chosen but have no real input or power in the research process. "Compliance": where outsiders decide the research agenda and direct the process, with tasks assigned to the participants and incentives being provided by researchers. "Consultation": where local opinions are sought but outside researchers conduct the work and decide on a course of action. "Co-operation": where local people

work together with outside researchers to determine the priorities of the research objective, with the responsibility remaining with outsiders for directing the process. “Co-learning”: where local people and outsiders share their knowledge to develop a new understanding and work together to form action plans, with outsiders providing the facilitation. “Collective action”: where local people set their own agenda and carry out the research in the absence of outside initiators and facilitators
